# Intoxication par la paraphényléne-diamine (takaout) au Maroc: à propos de 24 cas

**Published:** 2011-03-09

**Authors:** Ali Derkaoui, Smael Labib, Sanae Achour, Hicham Sbai, Mustapha Harrandou, Mohammed Khatouf, Nabil Kanjaa

**Affiliations:** 1 CHU Hassan II, Service d’Anesthésie Réanimation Fès Maroc; 2 CHU Hassan II, Laboratoire de Toxicologie Médicale et d’Analyse Toxicologique, Fès, Maroc, Faculté des Sciences et Techniques, Kenitra, Maroc

**Keywords:** Intoxication, paraphénylène-diamine, rhabdomyolyse, mortalité, Maroc

## Abstract

La paraphénylène-diamine (PPD) est une amine aromatique dérivée de l’aniline, utilisée depuis 1863 par les femmes dans un but cosmétique comme teinture capillaire noire ou adjuvant de henné dans plusieurs pays d’Afrique et de Moyen Orient. Le but de notre travail était de décrire les caractéristiques cliniques, paracliniques et évolutives de nos patients et de les comparer avec les données de la littérature. Il s’agissait d’une étude rétrospective portant sur les cas admis en réanimation (2003-2010). Les critères d’inclusion étaient d’ordre clinique, paraclinique, thérapeutique et évolutif. Durant la période de l’étude 24 patients ont été inclus provenant de la région de Fés-Boulmane. L’intoxication à la (PPD) représentait 26% de l’ensemble des intoxications admises au cours de la même période. L’âge moyen était de 23,6 + /- 11,6 ans. Il existait une prédominance féminine avec un sex-ratio de 4,7. L’intoxication était volontaire dans 82,6 %, accidentelle dans 8,6 %, et criminelle dans 4,3%. Le syndrome de rhabdomyolyse caractéristique de cette intoxication était retrouvé chez 60% de nos patients, l’atteinte respiratoire chez 56,5%, l’atteinte cardiaque était présente dans 30% des cas et 17,4% des patients avaient présentés une insuffisance rénale. La prise en charge thérapeutique se basait sur l’apport volémique massif, alcalinisation des urines ainsi que l’administration de corticoïdes et de diurétiques. Le recours à une trachéotomie de sauvetage était nécessaire chez 7 patients. Trois de nos patients avaient bénéficié d’une épuration extra rénale. L’évolution était fatale chez 47,8% des cas. La PPD représente ainsi la principale cause de mortalité toxique dans notre contexte. L’intoxication à la PPD, représente la première cause de rhabdomyolyse toxique dans notre contexte. Elle est responsable d’une mortalité très élevée. Ce qui implique une réglementation du commerce de la PPD, une information du corps soignant et une prise en charge précoce.

## Introduction

La paraphénylène-diamine (PPD) ou para-aminobenzène est une amine aromatique dérivée de l’aniline utilisée depuis longtemps par les femmes dans un but cosmétique comme teinture capillaire noire ou adjuvant de henné dans plusieurs pays d’Afrique et du Moyen Orient. Cette substance chimique est très connue dans l’industrie occidentale depuis un demi-siècle sous de nombreux noms: paramine, fouramine D, ursol D, vulpa D, furol S, comme teinture des fourrures, dans la fabrication d’articles domestiques, d’agents cosmétiques, des pneus, et le développement photographique [1].

Au Maroc, elle est librement vendue chez les herboristes traditionnels sous le nom de Takaout Roumia par analogie à un produit végétal connu sous le nom de Takaout Beldia.

La large utilisation de la PPD et sa disponibilité en absence de réglementation de son emploi ont conduit à la découverte de ses effets toxiques, d’où son usage fréquent dans un but d’autolyse. En effet, absorbée par voie orale, elle est responsable d’intoxication grave réalisant en premier temps une détresse respiratoire qui met en jeu le pronostic vital, associée à une rhabdomyolyse et à une insuffisance rénale aigue [2].

L’intoxication à la PPD est devenue un véritable problème de santé au Maroc puisqu’elle est responsable d’une mortalité importante chez des sujets jeunes [3-4].

Ce travail est une étude rétrospective ayant concerné tous les dossiers de patients admis au service de réanimation au CHU Hassan II de Fès pour intoxication à la PPD durant une période de 7 ans. Le but de notre travail était de décrire les caractéristiques cliniques, paracliniques et évolutives de nos patients et de les comparer avec les données de la littérature.

## Méthods

Il s’agit d’une étude rétrospective effectuée sur une période de 7ans; du mois de Janvier 2003 au mois de Décembre 2010 et incluant 24 patients pris en charge dans le service de réanimation polyvalente du CHU Hassan II de Fès (Maroc) pour intoxication à la paraphénylène-diamine. Les paramètres épidémiologiques et les données cliniques, paracliniques, thérapeutiques et évolutifs de l’intoxication ont été analysés pour chaque cas. Les résultats ont été exprimés en pourcentage ou en moyenne ± écart-type

## Résultats

**Figure 1:**
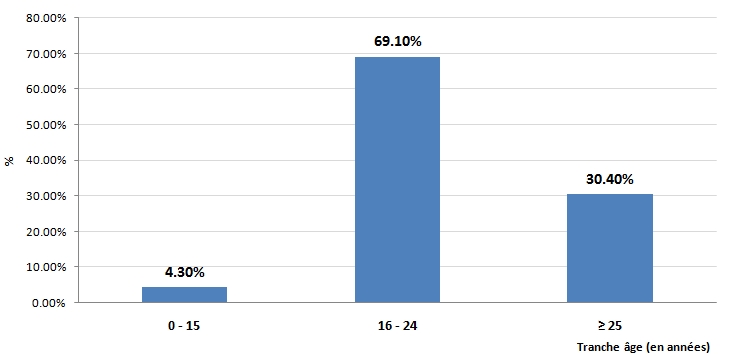
Répartitions des intoxications à la paraphénylène-diamine en fonction du groupe d’âge

Nous avons recensé 24 patients durant la période d’étude. L’âge moyen des patients était de 23,6 (± 11 ans), avec des extrêmes allant de 4 ans à 41 ans. La tranche d’âge la plus touchée était comprise entre 16 et 24 ans (65,2%). Deux cas pédiatriques ont été recensés (enfants ayant ingérés le produit de manière accidentelle) (Figure 1).

Le sexe féminin était largement prédominant soit (82%, sex-ratio de 4,7). Des antécédents psychiatriques à type de dépression ou de psychopathie étaient retrouvés dans 8,6% des cas.

Le délai moyen de prise en charge était de 4,28 (± 3,13 heures) avec des extrêmes allant de 1 à 8 heures.

**Figure 2:**
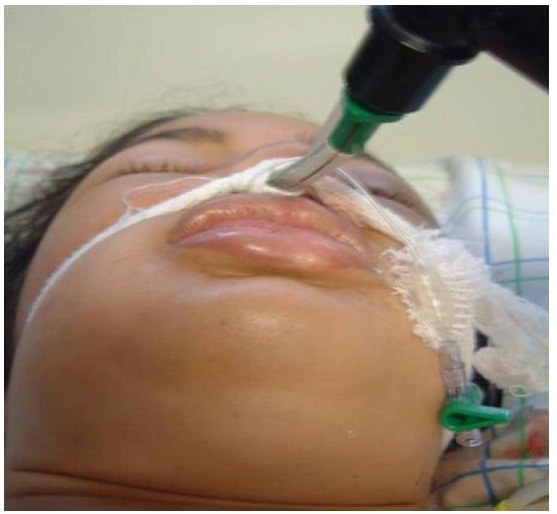
Œdème cervico-faciale chez une patiente marocaine après ingestion de paraphénylène-diamine

**Figure 3:**
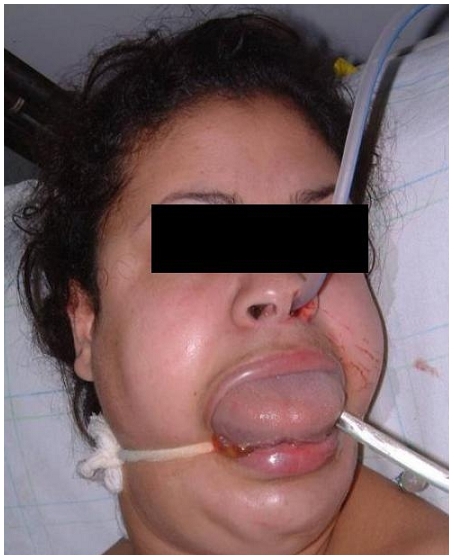
Macroglossie chez une patiente marocaine après ingestion de paraphénylène-diamine

La symptomatologie des intoxications aigues par la PPD est stéréotypée, le tableau clinique était dominé au départ par un oedème cervico-facial avec une atteinte respiratoire puis par un tableau musculaire. L’installation précoce d’un oedème typique de la face et du cou était notée chez 56,5% des patients engendrant ainsi une détresse respiratoire à l’admission, avec une fréquence respiratoire moyenne de 29,3 (± 4,6) cycles respiratoire/ mn. Une cyanose était retrouvée dans 8,6% des cas. Une atteinte musculaire était présente dans 60,8% cas à type de myalgies avec impotence fonctionnelle; 17,4% des cas ont présenté un oedème musculaire (Figure 2,Figure 3).

Des signes digestifs mineurs ont été notés telles des douleurs épigastriques et des vomissements dans 13 cas (56,5 %).

L’aspect des urines était porto chez tous les patients. La diurèse moyenne recueillie était de 1,8 (± 0,8 l/24/h; de 400 ml à 3,2 l/j). Une oligoanurie (diurèse inferieure à 500 ml/24 h) était observée dans 8,7% des cas.

La majorité des patients avaient un bon état hémodynamique, la pression artérielle systolique (PAS) était en moyenne de 117,1 (± 23,2 mmHg). 3,2% des intoxiqués étaient admis dans un état de choc (PAS inférieure à 80 mmHg, pâleur, extrémités froides, sueurs, troubles de la vigilance). Le score de Glasgow moyen à l’admission était de 12,11 (± 3,8) avec des extrêmes allants de 7à15. 8,6% de nos patients avaient présentés des crises convulsives avec un syndrome confusionnel. Une agitation psychomotrice était notée dans 4,3% des cas.

L’ionogramme réalisé chez tous les patients intoxiqués montrait une intégrité de la fonction rénale chez 82,6% des cas et une insuffisance rénale dans 4 cas (17,4%). Nous avons noté également une hyperkaliémie chez 17,4% des cas, une hyperglycémie chez 8 patients (34,7%) et une hypocalcémie chez 2 patients (8,7%). On a noté également une élévation des enzymes sériques d’origine musculaire (principalement CPK) mettant en évidence l’importance de la lyse musculaire, avec une augmentation des taux des CPK chez 21,7% des patients. Le taux des troponines Ic était élevé (> 0,1) chez 7 patients.

L’électrocardiogramme avait montré des anomalies électriques chez 60,8 % des patients. Ces anomalies étaient dominées par des troubles de rythme dans 40 % des cas à type d’extrasystoles ventriculaires, des troubles de conduction dans 13% des cas à type de blocs de branches droit et gauche et des troubles de la repolarisation à type onde T négative dans 8,6 % des cas.

Sur le plan radiologique, 8,7 % des patients ont présenté des images d’inhalation sur la radiographie pulmonaire, un pneumothorax chez 4,3 % des patients, un oedene aigue du poumon (OAP) lésionnel chez un patient. Elle était normale dans 82,6% des cas.

La prise en charge thérapeutique était essentiellement symptomatique associée aux mesures de décontamination. Tous nos patients ont bénéficié d’un lavage gastrique à l’admission aux urgences. La prise en charge thérapeutique de la détresse respiratoire a consisté en une ventilation mécanique (VM) chez 40% des patients, dont 29 % étaient trachéotomisés dès l’admission devant l’intubation difficile du fait de l’importance de l’oedème cervico-facial. La durée moyenne de ventilation était de 4 (± 5,5) jours avec un minimum d’un jour et un maximum de 10 jours. Tous les patients ont bénéficiés d’un remplissage vasculaire abondant avec du sérum salé isotonique ainsi que d’un traitement diurétique avec une alcalinisation dans le but d’obtenir un PH urinaire > 7. Un support hémodynamique était nécessaire dans 13% des cas. L’hémodialyse a été réalisée chez 13% des patients devant une hyperkaliémie (8,6%) et/ou effondrement de la diurèse (5%), en moyenne 2 séances avec des extrêmes d’une à quatre séances.

La durée moyenne de l’hospitalisation était de 3 (± 5) jours avec des extrêmes allant de 1 à 20 j. Le taux de létalité était très lourd (47,8%), dont la principale cause était le choc cardiogénique dans 13% suivi du choc septique dans 8,6 %.

## Discussion

Le marché marocain s’est approprié depuis 1970, d’un produit minéral à base de PPD qui a détrôné la Takaout commune beldia non seulement pour ses qualités tinctoriales remarquables, mais aussi pour sa grande disponibilité à petit prix et ses qualités de produit d’importation ce qui lui a valu l’appellation par extension de Takaout Roumia [3]. La toxicité expérimentale de la PPD est connue depuis longtemps, les premiers tests sont réalisés par Dubois et Vignon en 1898, alors que les cas d’intoxication humaine sont rares en occident. En 1924, Nott a décrit le premier cas d’intoxication systémique à la PPD chez un propriétaire de salon de coiffure suite au maniement de la teinture capillaire. Depuis, d’autres cas sont rapportés [3-4]. Au Maroc, le premier cas rapporté date de 1978 et depuis plusieurs publications se sont succédé [5-6]. Depuis les années 1980, on note une augmentation importante de cette intoxication. Moutaouakkil [2], avait publié une série de 315 patients colligés sur 5 ans au CHU de Casablanca avec un âge moyen de 23 ans, une prédominance féminine (91%) et la prise dans un but d’autolyse dans 93%.

Le présent travail confirme cette tendance, puisque l’âge moyen de nos patients était de 23,6 ans et le sexe féminin est majoritaire (82%) (Tableau 1 et 2).

Cette intoxication reste parmi les plus fréquentes en réanimation en milieu marocain comparativement au pays européen où les intoxications médicamenteuses restent les plus fréquentes [7]. Dans notre étude elle représente 26% de l’ensemble des intoxications aigues tout produit confondu. L’intoxication à la PPD peut être retrouvée à tout âge, mais la population jeune reste la plus touchée.

Pour la plupart des cas, l’intoxication est secondaire à la prise du toxique par voie orale, et pour la plupart des études l’ingestion est faite dans un but d’autolyse. En plus de son utilisation à des fins suicidaires, criminelles ou même abortives, on rapporte également son utilisation comme remède contre la douleur par Averbukh [8], ou contre la constipation par Schemesh [9], mais l’utilisation à des fins autolytique reste toujours prédominante.

Le tableau clinique de l’intoxication à la PPD est le suivant: après un intervalle libre d’en moyenne deux heures, s’installe un tableau clinique stéréotypé fait essentiellement d’une atteinte respiratoire mettant en jeu le pronostic vital dans l’immédiat et d’une atteinte musculaire et rénale pouvant le compromettre secondairement. Le tableau clinique initial comporte une sensation de brûlures bucco-pharyngées, une sialorrhée, des vertiges, des nausées, des vomissements, des épigastralgies, le tout parfois accompagné d’un trismus et des myalgies souvent intenses. Puis apparaît un oedème chaud, dur, douloureux et prurigineux intéressant d’abord la langue responsable de la macroglossie, les lèvres puis la région cervico-faciale. Rapidement le pharynx et les voies aériennes supérieures sont atteints avec installation d’emblée d’une dyspnée voire même une détresse respiratoire aigue. L’atteinte respiratoire constitue un facteur pronostique majeur. Allant d’une simple dyspnée à une détresse respiratoire aiguë. Cette atteinte fait intervenir plusieurs facteurs [6,10-12] : premièrement l’oedème cervico-facial précoce s’étendant au larynx et aux voies respiratoires supérieures est à l’origine d’un syndrome asphyxique menaçant le pronostic vital et imposant le recours en urgence à une intubation trachéale voire une trachéotomie de sauvetage; secondairement, la rhabdomyolyse des muscles respiratoires notamment le diaphragme; troisièmement la méthémoglobinémie aggravant l’hypoxémie déjà existante. Dans notre série, le dosage de cette dernière n’a pas été fait. Cette détresse respiratoire a été observée chez 56,5 % de nos patients.

La rhabdomyolyse réalise un syndrome clinique et biologique secondaire à la lyse des fibres musculaires striées ayant pour conséquence la libération dans le sang de myoglobine, d’enzymes et d’électrolytes. Le tableau clinique est fait essentiellement d’un syndrome musculaire et d’un syndrome urinaire [13,14]. Le syndrome musculaire fait de myalgies spontanées ou provoquées avec impotence fonctionnelle touchant les membres et une faiblesse ou fatigabilité. Les muscles atteints sont durs, tendus et sensibles à la palpation. L’apparition d’un myxoedème se traduit par un gonflement douloureux localisé ou généralisé. Une myolyse généralisée peut simuler une quadriplégie. Le syndrome urinaire est constant mais transitoire caractérisé par l’existence d’une myoglobinurie marquée par des urines noirâtres. L’élévation de la concentration sérique de la créatine-phosphokinase (CPK) affirme le diagnostic de la rhabdomyolyse. Une concentration au moins supérieure à cinq fois la valeur normale est nécessaire pour retenir le diagnostic. Il existe un parallélisme entre l’intensité de la lyse musculaire et le niveau d’élévation des CPK [15]. Dans notre série, le taux des CPK était en moyenne 4881,9 UI/l.

L’insuffisance rénale myoglobinurique évolue en deux phases, une phase oligoanurique ou anurique et une phase de reprise de la diurèse avec normalisation de la fonction rénale à partir de la troisième semaine. L’atteinte rénale est de type nécrose tubulo-interstitielle aiguë caractéristique des insuffisances rénales aiguës accompagnant les rhabdomyolyses [16,17]. Dans notre série, malgré le traitement agressif de l’hypovolémie, le recours au remplissage abondant et l’alcalinisation systématique de même que l’utilisation précoce des diurétiques, 3 patients sur 24 ont présenté une anurie ayant nécessité le recours à l’hémodialyse. Ce taux de survenue d’IRA anurique est relativement faible en comparaison avec les autres séries de la littérature [2]. Le retard de prise en charge thérapeutique est certainement un élément déterminant dans la survenue d’une atteinte rénale.

L’atteinte cardiaque réalise un tableau de myocardite aiguë par atteinte directe du myocarde, analogue à l’atteinte des muscles squelettiques. Cette atteinte se traduit par une baisse globale de la contractilité des deux ventricules et la constitution d’un thrombus intra-ventriculaire. L’autopsie montre des altérations diffuses faites de congestion,d’homogénéisation et de marginalisation des noyaux, ces lésions sont associées parfois à une dystrophie ou à une myocardite interstitielle. Le dosage de la Troponine permet le diagnostique précoce du dommage myocardique [18,19].

On peut observer des signes d’accompagnements bénins: céphalées, vertiges, asthénie intense, tachycardie, polypnée. Dans les formes graves; peut survenir un coma souvent précédé de phase de somnolence entrecoupée d’agitation.

L’analyse toxicologique a un intérêt diagnostique certain, mais elle n’est pas indispensable lorsque le diagnostique est évident cliniquement. Cette analyse toxicologique doit être effectuée sur le contenu gastrique, le sang, les urines et le liquide pleural.

Le traitement est uniquement symptomatique. À l’heure actuelle, il n’y a pas d’antidote. Les différents traitements ont pour objectif de diminuer la charge du produit dans le corps des victimes, et de combattre les manifestations de l’intoxication, notamment celles qui pourraient engager le pronostic vital.

Le lavage gastrique doit être entrepris en urgence, même au-delà de la deuxième heure après l’absorption. Il doit être fait dans des conditions de sécurité parfaite et dans le strict respect des contre-indications. Il doit être abondant jusqu’à éclaircissement du liquide. Le prélèvement initial de liquide de lavage gastrique sera adressé pour analyse toxicologique. La PPD, comme les dérivés aminés aromatiques, est bien absorbée sur le charbon actif. Ce dernier peut aider à diminuer la charge du toxique et présente l’avantage d’être facile à administrer et d’être sans effets secondaires [7]. Malheureusement à l’heure actuelle ce produit n’est pas disponible dans les hôpitaux marocains.

La PPD n’est pas dialysable. L’espace de diffusion de la PPD est très large, il intéresse les parenchymes musculaires, la forme libre est négligeable par rapport à la forme liée, l’épuration extra rénale est donc inopérante.

Le plus important en début de prise en charge des intoxiqués en milieu de réanimation est d’assurer la liberté des voies aériennes supérieures, ce qui nécessite le recours à l’intubation endotrachéale et à la ventilation artificielle [2,20]. En cas d’échec de l’intubation, à cause de l’oedème cervico-facial, le recours à la trachéotomie de sauvetage dans ce contexte, constitue la seule alternative pour le traitement de la détresse respiratoire.

La réanimation volémique repose sur l’administration précoce et massive de solutés de perfusion, visant à lutter contre l’hypovolémie constante liée à la rhabdomyolyse, et au besoin une alcalinisation des urines. Le remplissage vasculaire, doit être précoce, massif et rapide. Il permet d’augmenter le débit tubulaire et la pression de perfusion rénale favorisant d’une part l’élimination de toutes les substances capables de se précipiter et d’obstruer la lumière tubulaire et d’autre part, la prévention contre le passage à l’organicité de l’insuffisance rénale fonctionnelle secondaire à l’hypovolémie. L’hyperdiurèse alcaline se base sur l’apport de sérum bicarbonaté à 0,14% qui vise à maintenir un pH urinaire supérieur à 7 pour favoriser l’élimination de la myoglobine qui ne se fait qu’en milieu alcalin. Cette diurèse osmotique alcaline doit être maintenue jusqu’à la disparition de la myoglobinurie (survenant habituellement au 3ème jour). En l’absence de reprise de la diurèse après la restauration d’une volémie efficace et d’un équilibre hydro-éléctrolytique satisfaisant, il faut avoir recours aux diurétiques [21].

L’epuration extra rénale est indiquée devant une oligoanurie persistante malgré une réanimation adéquate ou bien devant une hyperkaliémie menaçante.

A partir du 2ème jour d’évolution de la rhabdomyolyse, le risque de compression vasculaire et nerveuse est important surtout si la myolyse est entendue. L’indication de l’aponévrotomie de décharge sera posée chaque fois qu’il existe des signes de compression vasculaire ou nerveuse dépistés [22].

Le traitement de la méthémoglobinémie se fait par administration de vitamine C ou de bleu de méthylène. L’acide ascorbique agit par réduction directe de la méthémoglobine en hémoglobine, jusqu’à 4 g/jour. En cas d’inefficacité de la vitamine C, le bleu de méthylène parait indispensable, la dose préconisée est de 1 à 3 ml/kg de poids, ce qui correspond à la perfusion d’une ampoule de 20 ml à 1 % dans 250 ml de sérum glucosé à passer en une heure [23].

La mortalité des intoxications à la PPD est très lourde dans toutes les séries, elle varie de 20 à 42 %. Elle est liée au retard de consultation dû surtout à l’éloignement géographique par rapport à la structure hospitalière, au bas niveau socioéconomique, enfin, à la méconnaissance des signes cliniques et des bases thérapeutiques de ces intoxications par le personnel médical comme en témoignent les prescriptions aléatoires souvent insuffisantes surtout au service des urgences. Les causes de mortalité sont généralement le syndrome asphyxique à la phase initiale et la rhabdomyolyse et ses complications électrolytiques et rénales à la phase secondaire. Le pronostic dépend de plusieurs facteurs qui sont [5,15]: la dose ingérée, le délai de la prise en charge, l’existence ou non de signes cliniques ou électriques en faveur d’une atteinte myocardique et l’existence ou non d’une insuffisance rénale aiguë anurique. Au Maroc, ces intoxications sont fréquentes, graves, et souvent mortelles.

## Conclusion

L’intoxication à la PPD est un véritable problème de santé au Maroc. Elle réalise une affection grave dominée par la détresse respiratoire à l’origine de la plupart des décès primaires. Elle concerne dans notre contexte surtout des femmes jeunes qui ingèrent le produit dans un but d’autolyse. Le diagnostic est surtout clinique et le traitement purement symptomatique à défaut d’antidote. Le pronostic est sombre, la mortalité est élevée, de ce fait, elle doit être considérée comme une urgence diagnostic et thérapeutique. La disponibilité commerciale de ce produit sous sa forme pure devient inquiétante, justifiant le recours à un large programme de prévention pour informer le public et les autorités du danger de la PPD. L’interdiction de l’importation et de la vente de la PPD pure ou en combinaison avec d’autres teintures et la réglementation de son utilisation industrielle est urgente. Sur le plan médical, les intoxiqués doivent être pris en charge immédiatement dans les services de réanimation et les professionnels de santé ont besoin d’être familiarisés avec le tableau clinique et le danger potentiel de cette intoxication.

## Conflits d’intérêt

Les auteurs ne declarent aucun conflit d’interêts

## Contribution des auteurs

Tous les auteurs ont également contribué à ce travail et ont lu et approuvé la version finale du manuscrit.

## Figures and Tables

**Table 1:** Incidence de l’intoxication à la paraphénylène diamine au Maroc de 1988 à nos jours; revue de la littérature de 1988 a nos jours

**Auteurs**	**Année**	**Région**	**Nombre de cas**
Bourquia^5^	1988	Casablanca	4
Jrouni^24^	1992	Agadir	56
Zeggwagh^18^	1996	Rabat	95
Kamga^25^	1998	Casablanca	61
Benchama^26^	2001	Oujda	31
Moutaouakkil^2^	1999-2001	Casablanca	315
Notre série	2003-2010	Fès	24

Références

5. A Bourquia, AJ Jabrane, B Ramdani, D Zaid. Toxicité systémique de PPD. Presse Med. 1988 Oct 15;17(35):1798-800.

24. T Jrouni. Intoxication à la PPD dans la région d’Agadir (56 cas). Thèse de doctorat en médecine, Rabat. 1992; (262).

18. Zeggwagh AA, Abouqal R, Abidi K, Madani N, Zekraoui A, Kerkeb O. Thrombus ventriculaire et myocardite toxique par la PPD. Ann Fr Anesth Reanim. 2003 Jul;22(7):639-41.

25. Kamga Nelly Murielle. Intoxication aigue par la PPD (61 cas). Thèse de doctorat en médecine, Casablanca. 1998; (43).

26. N Benchama. Les intoxications aigues par la PPD à l’hôpital Al Farabi à oujda. Thèse de doctorat en médecine, Casablanca. 2001; (19).

2. S Moutaouakkil, B Charra, A Hachimi, H Ezzouine, H Guedari, H Nejmi, A Benslama. Rhabdomyolyse et intoxication à la paraphénylènediamine. Ann Fr Anesth Réanim. 2006; (25) :708-713.

**Table 2:** Pourcentage de la population féminine dans l’intoxication à la paraphénylène diamine au Maroc de 1988 à nos jours, revue de la littérature de 1988 à nos jours

**Auteurs**	**Nombre de cas**	**Pourcentage des femmes (%)**
Atlassi^27^	4	100
Jrouni^24^	56	80,35
Fatihi^16^	13	92,30
Kamga^25^	61	95,08
Yagi^1^	18	95,08
Moutaouakkil^2^	315	91
Notre série	24	82

Références

27. M Atlassi. Intoxication par takaout roumia ou PPD (4 cas). Thèse de doctorat en médecine, Casablanca. 1988; (225)

24. T Jrouni. Intoxication à la PPD dans la région d’Agadir (56 cas). Thèse de doctorat en médecine, Rabat. 1992; (262)

16. E Fatihi, M Laraki. Toxicité systémique de PPD (13 observations). Reanim Urg. 1995;(4):371373

25. Kamga Nelly Murielle. Intoxication aigue par la PPD (61 cas). Thèse de doctorat en médecine, Casablanca. 1998; (43)

1. H Yagi, AM El Hendi. Acute poisoning from hair dye. East Afr Med J. 1991; (68):404

2. S Moutaouakkil, B Charra, A Hachimi, H Ezzouine, H Guedari, H Nejmi, A Benslama. Rhabdomyolyse et intoxication à la paraphénylène-diamine. Ann Fr Anesth Réanim. 2006; (25) :708-713
